# Bimodal Poly(lactic-co-glycolic acid) Nanocarrier with Zinc Oxide and Iron Oxide for Fluorescence and Magnetic Resonance Imaging

**DOI:** 10.3390/molecules30081818

**Published:** 2025-04-18

**Authors:** Thúlio Wliandon Lemos Barbosa, Laurent Lemaire, Isabelle Verdu, Larissa Santos, Natália Galvão de Freitas, Mariana Picchi Salto, Leila Aparecida Chiavacci

**Affiliations:** 1 School of Pharmaceutical Sciences, São Paulo State University (UNESP), Araraquara 14800-903, SP, Brazil; l.santos@unesp.br (L.S.); n.freitas@unesp.br (N.G.d.F.); mariana.psalto@gmail.com (M.P.S.); 2 National Institute of Health and Medical Research (INSERM), National Centre for Scientific Research (CNRS), Materials, Engineering, Nanosciences, and Technologies (MINT), Research and Training System-Interactions, Catalysis, Applications, and Technologies (SFR ICAT), University of Angers, F49000 Angers, France; laurent.lemaire@univ-angers.fr (L.L.); isabelle.verdu@univ-angers.fr (I.V.); 3 Platform for Research in Imaging and Multimodal Spectroscopy (PRISM), SFR ICAT, University of Angers, F49000 Angers, France

**Keywords:** contrast agents, zinc oxide, iron oxide, poly(lactic-co-glycolic acid), fluorescence imaging, magnetic resonance imaging

## Abstract

Zinc oxide (ZnO) and iron oxide (IO) nanoparticles have been identified as promising candidates for biomedical applications, based on their unique physicochemical properties. The association of these nanoparticles in a single system creates a bimodal entity, allowing the excellent luminescent properties of ZnO quantum dots to be combined with the contrast agent of IO for magnetic resonance imaging (MRI). The present study focuses on the luminescent and MRI properties of a new poly(lactic-co-glycolic acid) (PLGA) nanocarrier system formulation containing ZnO NPs and IO NPs in different nominal ratios. Microscopic analysis (TEM and SEM) reveals a circular morphology with IO and ZnO NPs. The average diameter of the particles was determined to be 220 nm, as measured by DLS. The luminescence results indicate that the PLGA system shows strong emission in the visible range, and the MRI analysis shows a high *r2* relaxivity of 171 mM^−1^ s^−1^ at 7T. The optimized formulation, exhibiting a molar ratio of Fe:Zn ranging from 1:10 to 1:13 (mol:mol), demonstrates superior fluorescence and MRI performance, underscoring the significance of nanoparticle composition in bimodal imaging applications. The systems evaluated demonstrate no toxicity in the THP-1 cells for doses of up to 128 µg mL^−1^, with efficient labeling after 4 h of incubation, yielding images of strong luminescence and T2 contrast. The PLGA:ZnO:IO system demonstrates considerable potential as a bimodal platform for diagnostic imaging.

## 1. Introduction

Quantum dots (QDs) or semiconductor materials stand out for their ability to produce high-resolution images due to their unique luminescent and electrical properties resulting from quantum confinement. QDs are particularly useful in diagnostic imaging, due to their ability to enhance image contrast through fluorescence, in combination with dedicated contrast agents for magnetic resonance imaging (MRI) [1,2,3,4,5]. They have also been used in photodynamic therapy [6,7,8], controlled drug delivery [9,10,11], and cell markers [12,13]. 

The toxicity of QDs is dependent on their physicochemical properties and environmental conditions, such as charge, size, concentration, and surface coating [14]. If the QDs’ core is based on metals such as cadmium and selenium, which have high quantum yields, their use is limited as they are toxic (e.g., CdTe QDs and CdSe/ZnS QDs) [14,15,16]; this does not seem to be the case for zinc-based QDs.

Zinc oxide is a semiconductor employed frequently due to its biocompatibility, chemical stability, biodegradability, biosafety, and excellent optical and electrical properties. Its versatility enables applications in chemical detection, biosensors, photocatalysts, and other biomedical uses [17]. Its characteristics include a wide band gap of 3.37 eV and a significant exciton binding energy of 60 meV at room temperature (RT), which contribute to its efficient light absorption and emission properties [18,19]. Furthermore, research has been conducted on its application in the domain of fluorescence imaging for the detection of tumor cells, resulting in a high fluorescence yield [17,20,21]. 

MRI techniques can also employ nanoparticle-based contrast agents, such as superparamagnetic iron oxide nanoparticles (SPIONs). SPIONs, in their magnetite form, exhibit superparamagnetic properties that make them responsive to the external magnetic fields used in MRI [22]. This enables a strong interaction with the magnetic fields of the MRI equipment, resulting in a high-contrast image signal. In addition to its magnetic properties, iron oxide finds application in cancer treatment through magnetic hyperthermia, nanocatalysis, and the generation of reactive oxygen species to induce cancer cell apoptosis [23].

These two compounds, iron oxide nanoparticles (IO NPs) and zinc oxide nanoparticles (ZnO NPs), are interesting due to their unique physicochemical properties, low cost, and the non-toxicity of the zinc and iron elements [24]. IO stands out for its magnetic properties [25,26,27,28], and the ZnO NP is known for its luminescent properties [29,30,31,32]. However, studies investigating the concomitant use of ZnO and IO as bimodal imaging agents are limited [33,34]. In Gupta et al. [33], a core (IO) shell (ZnO) structure is reported, along with its biocompatibility and capacity to label cells. In Lai et al. [34], research was carried out on the in vivo biosynthesis of ZnO and IO nanoclusters in Alzheimer models. Indeed, after the administration of iron(II) chloride and zinc gluconate, the formation and accumulation of metallic nanoclusters in the brains of mice were detected using fluorescence and magnetic resonance imaging.

**Figure 1:**
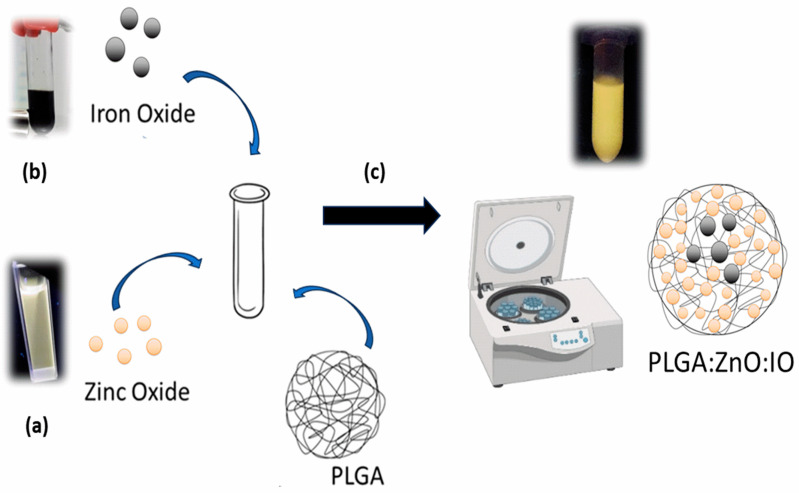
Synthesis scheme of PLGA particles containing ZnO NPs and SPIONs. (**a**) sol-gel method for ZnO NPs, adapted from Spanhel and Anderson [47]; (**b**) co-precipitation method for IO NPs, adapted from Babes et al. (1999) [48]; and (**c**) PLGA particles produced by a simple solvent evaporation emulsion reaction, adapted from Luque-Michel et al. (2021) [45].

To the best of our knowledge, no studies have proposed a bimodal imaging system based on ZnO and IO within a polymeric system, such as poly(Lactic-co-Glycolic Acid) (PLGA), which promotes stability and biocompatibility, while maintaining reduced toxicity of inorganic compounds and promoting vectorization for specific targeting, with potential application as a theranostic drug [35]. However, there are reports on ZnO-encapsulated PLGA, especially for antimicrobial activity [36,37,38,39,40], and IO-encapsulated PLGA for photodynamic therapy and drug delivery for diseases such as cancer [41,42,43,44,45,46]. The objective was to develop a biocompatible and stable PLGA system that contains both nanoparticles, maintaining their fluorescence and magnetic properties to provide a bimodal contrast agent (Figure 1). A simple emulsion and solvent evaporation method was developed, and an extensive study was conducted to optimize the system. The interaction between the two nanoparticles, based on their magnetic and luminescent properties, was investigated. Finally, a study assessed the cellular labeling and potential toxicity. This study utilized high-quality images via fluorescence and MRI to provide comprehensive insights into the system’s performance.

## 2. Results and Discussion

The nominal ratio between PLGA, ZnO, and IO was assessed to ensure that the resulting product exhibits favorable luminescent and magnetic characteristics. For instance, a sample with a nominal PLGA:ZnO:IO ratio of 10:1:1 (*w*:*w*:*w*, mg) indicates the intended use of 10 mg PLGA, 1 mg ZnO, and 1 mg IO.

During the experiments, interactions between ZnO, iron oxide, and PLGA influenced the luminescence and magnetic intensity of the materials. These interactions were analyzed, providing insights into the system’s stability.

### 2.1. Stability Assessment of the PLGA System: Hydrodynamic Diameter Distribution Analysis

The particle size distribution of the PLGA systems was determined using the intensity-weighted mean hydrodynamic diameter (d.nm) (Table 1). The stability in the PVA and T80 surfactant systems, at concentrations of 0.4% and 0.3%, respectively, was evaluated on the day of synthesis and 15 days post-synthesis.

The diameter of PLGA-IO NPs with varying IO ratios exhibited no significant changes following a 15-day storage, as indicated in Table 1. Additionally, the hydrodynamic diameter of the PLGA:IO particles exhibited uniformity across varying IO ratios, with an average measurement of 150 nm. Particle uniformity was corroborated by the polydispersity index (PDI) values. Even after a 15-day storage period at 4 °C, the particles exhibited stability in suspension. The observed diameter of PLGA particles falls within the optimal range of 100 to 300 nm for effective cell incorporation [49].

The average diameter of PLGA:ZnO particles is approximately 220 nm. This diameter is greater than that of PLGA:IO particles (~150 nm), with a statistically significant difference (*p* < 0.05). This observation indicates that there are distinct interaction behaviors associated with the loading of ZnO NPs into PLGA. The diameter remained consistent after 15 days of storage at 4° C, indicating robust stability under these conditions.

For PLGA:ZnO:IO, the diameter of the particles ranged from 190 to 250 nm; this is a range similar to that of PLGA:ZnO synthesized without IO (211 to 250 nm). Over a 15-day storage period at 4 °C, formulations with higher IO content in PLGA:ZnO:IO exhibited particle growth, possibly due to interparticle interactions leading to aggregation. The increase in IO NPs may promote aggregation, likely due to a decrease in surface charge between IO particles at neutral pH during the PLGA formulation process [50]. Consequently, it is essential to determine the appropriate IO NP concentration to prevent excessive aggregation.

On the other hand, when the amount of IO was kept constant and the amount of ZnO was increased, no significant increase in the diameter of the PLGA particles was observed. Unlike IO NPs, ZnO NPs remain stable when the suspension pH is in the range of 7–8 [51]. Furthermore, although an increase in NP concentration can influence the particle aggregation, the amount of ZnO used in the formulations was not sufficient to induce an increase in PLGA particle diameter.

### 2.2. Characterization of the PLGA System: Transmission Electron Microscopy (TEM) and Scanning Electron Microscopy (SEM) Analysis

**Figure 2:**
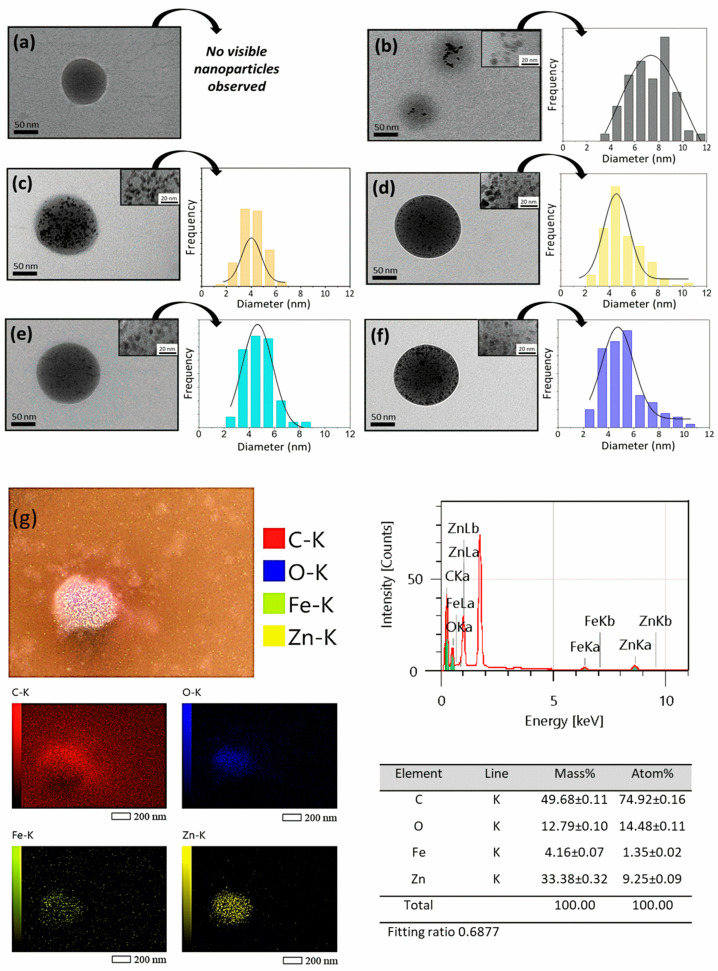
TEM images of PLGA particles (**a**–**f**). (**a**) PLGA unloaded; (**b**) PLGA:IO 10:1; (**c**) PLGA:ZnO 10:6; (**d**) PLGA:ZnO:IO 10:6:1; (**e**) PLGA:ZnO 10:8; and (**f**) PLGA:ZnO:IO 10:8:1. Gaussian distribution curve representing the NP diameter calculation based on the presence of ZnO and IO NPs as determined by TEM analysis; (**g**) SEM image of the PLGA:ZnO:IO 10:6:1 sample. Energy-dispersive X-ray spectroscopy (EDS) analysis is presented, showing the detection of the elements O-K, C-K, Zn-K, and Fe-K. The corresponding EDS spectrum and elemental mapping are displayed, confirming the distribution and composition of these elements within the sample.

The morphology of the NPs was analyzed by TEM, and the elemental analysis of iron and zinc elements in the PLGA systems was conducted by a combination of SEM and EDS analysis (Figure 2).

Additionally, PLGA particles containing only ZnO or IO NPs manifested as black dots, a phenomenon not observed in the image of unloaded PLGA. The diameter of the ZnO and IO NP was calculated by utilizing the Gaussian curve (Figure 2), and the resulting data are presented in Table 2.

The ZnO NPs present in the PLGA:ZnO 10:6 and 10:8 formulations have an average diameter of 4.73 nm and 4.56 nm, respectively. These results are consistent with those previously reported for ZnO NPs [52]. In the PLGA:IO 10:1 formulation, the IO NPs exhibited an average diameter of 7.33 nm, with the TEM analysis of isolated IO NPs revealing a comparable diameter of 8.07 nm (Figure S1).

Although there is no significant difference in the average diameters of the NPs observed in PLGA 10:8:1 or 10:6:1 (4.64 ± 0.14 nm and 4.03 ± 0.10 nm, respectively) compared to PLGA:ZnO 10:8 or 10:6 (4.73 ± 0.16 nm and 4.56 ± 0.16 nm, respectively), it can be noted that the formulations with IO (PLGA:ZnO:IO 10:8:1 and 10:6:1) exhibit a broader distribution, with NPs larger than 6 nm, which is consistent with the PLGA:IO 10:1 sample, while the formulations without IO (PLGA:ZnO 10:8 and 10:6) show a narrower distribution.

These results suggest that the NPs were successfully incorporated into the PLGA system. Elemental analysis through SEM images followed by EDS was performed to confirm the incorporation of ZnO and IO NPs. Furthermore, the integrated SEM and EDS analysis (Figure 2g) revealed that zinc and iron elements were predominantly situated within the PLGA:ZnO:IO particles, indicating their efficient incorporation into the polymeric matrix. These findings complement and reinforce the TEM results.

The surface charge was also studied using zeta potential (ZP) before the lyophilization (Table 2). The results indicate that the IO NP, in the presence of the ZnO NP, caused an increase in the zeta potential value of the PLGA particles. This increase in zeta potential may contribute to enhanced colloidal stability within the system, which can be attributed to the inhibition of particle aggregation.

### 2.3. PLGA Particles Incorporating Zinc Oxide and Iron Oxide Nanoparticles: Analysis of Luminescent Properties

**Figure 3:**
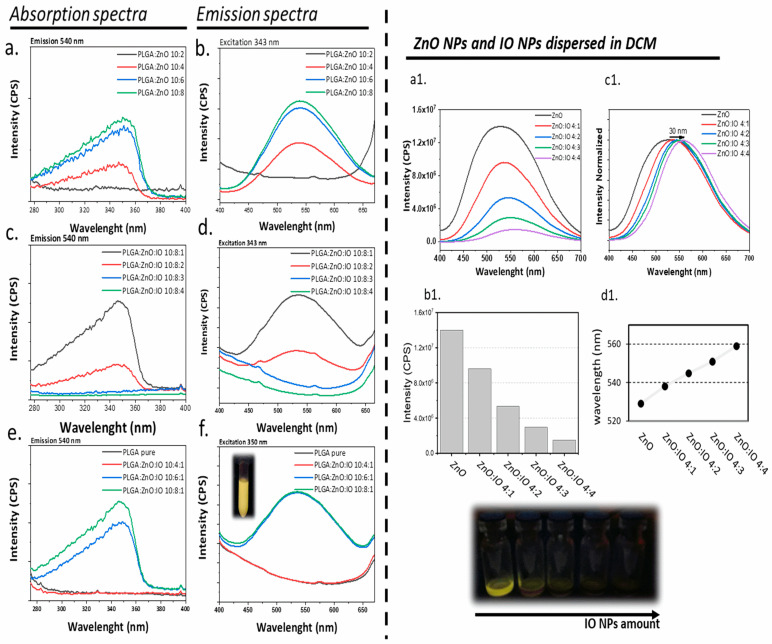
Fluorescence spectra of PLGA samples: absorption spectra and emission spectra for PLGA:ZnO samples (**a**,**b**), PLGA:ZnO:IO samples with the same quantity of ZnO and varying amounts of IO (**c**,**d**), and PLGA:ZnO:IO samples with the same quantity of IO and varying amounts of ZnO (**e**,**f**). The insets (**a1**–**d1**) highlight the fluorescence assay of ZnO NPs and IO NPs dispersed in dichloromethane (DCM), with a constant quantity of ZnO and varying amounts of IO: (**a1**) emission spectra upon excitation at 343 nm, (**b1**) maximum emission intensity as a function of IO concentration, (**c1**) normalized emission spectra, and (**d1**) maximum emission wavelength as a function of IO concentration.

The spectra demonstrating luminescence (emission and absorption) are presented in Figure 3. The luminescent properties of the PLGA system were adjusted based on the excitation (absorption) and emission spectra of pristine ZnO synthesized in previous work by our team [53]. The synthesized ZnO NP, with a diameter ranging from 3 to 4 nm, exhibits a peak emission at ~540 nm when excited at 343 nm. The excitation wavelength of ZnO typically varies around 350 nm, with a decrease in emission capability at longer wavelengths, generally starting from ~365 nm. This variation in the emission spectrum is attributed to the diameter of the produced ZnO NPs [54].

The behavior of the emission spectra varies with the concentration of ZnO in the PLGA polymer system. Therefore, 343 nm is a suitable excitation wavelength for evaluating ZnO luminescence, as it avoids excitation loss regardless of NP diameter or concentration. Notably, the luminescence intensity increases with increasing ZnO concentration in the polymer system (Figure 3b). The PLGA:ZnO 10:8 sample exhibits the highest luminescence intensity, although it is similar to PLGA:ZnO 10:6, suggesting a possible plateau in luminescence intensity. No luminescence was observed in the PLGA:ZnO 10:2 sample, and a low luminescence intensity of the PLGA:ZnO 10:4 sample was observed, despite several attempts, suggesting that there is a minimum concentration of ZnO required for the luminescent properties to manifest. Based on these observations, the minimum and maximum concentrations of ZnO and IO in the PLGA preparation were evaluated (Figure 3c–f).

If the amount of ZnO affects the luminescence intensity of PLGA systems, the presence and amount of IO also impact the luminescence, as shown in Figure 3c,d. PLGA:ZnO:IO 10:8:1 shows robust luminescence intensity. However, doubling the amount of IO in the formulation process to produce the PLGA:ZnO:IO 10:8:2 sample reduced the luminescence intensity. At higher IO amounts, luminescence becomes negligible. Consequently, the optimal IO nominal concentration for assessing luminescence in the current PLGA:ZnO:IO samples is determined by a quantity of 1 mg.

In the analysis of Figure 3e,f, where the ZnO concentration is varied while the IO concentration is kept constant, the emission remains constant for the PLGA:ZnO:IO 10:6:1 and 10:8:1 samples, but not for the PLGA:ZnO:IO 10:4:1 sample. This latter formulation was synthesized several times, consistently yielding the same result. The last one was synthesized multiple times, consistently producing the same result. Therefore, a minimum of 6 mg of ZnO is necessary to impart luminescent properties to this material when a nominal amount of 1 mg of IO is present in the PLGA particle preparation. An initial amount of ZnO NPs between 6 and 8 mg can ensure a luminescence plateau in samples containing 1 mg of IO.

Regarding the luminescent properties and ICP results of different Zn and Fe concentrations (Table 3), the quantified ZnO content for PLGA:ZnO:IO at 10:6:1 and 10:8:1 was 50.01 ± 0.34 mM and 45.57 ± 0.42 mM, respectively. These values indicate a saturation point for ZnO incorporation in the PLGA matrix, which is consistent with the observed luminescence intensity plateau. In formulations containing only ZnO, the luminescence intensity increased with the nominal ZnO concentration, as expected. However, in systems co-loaded with ZnO and IO, the presence of IO NPs interfered with the luminescent response. According to the ICP data, the IO:ZnO ratio shifted from 1:10 to 1:13 as the formulation changed from PLGA:ZnO:IO 10:6:1 to 10:8:1. While absorption intensity (Figure 3e) increased with higher ZnO content relative to IO, this variation had no significant effect on emission intensity at the excitation wavelength of 343 nm.

The results show that increasing the nominal concentration of IO significantly affects the material’s luminescent properties. To investigate the interaction between IO and ZnO NPs under controlled conditions, we conducted a study using different nominal concentrations of IO while keeping the nominal concentration of ZnO dispersed in DCM (Figure 3a1–d1). These results allow us to draw two important conclusions. First, an increase in IO concentration can reduce the luminescence intensity of the dispersion. Second, as the IO concentration increases, there is a 30 nm “red shift” in the maximum emission wavelength. Consequently, the luminescence properties of the ZnO NP can be influenced not only by its concentration in the PLGA particles, but also by the presence of the IO NP.

This phenomenon is attributed to a specific property of iron in fluorescent compounds known as the quenching effect, where the reduction in luminescence intensity occurs due to several factors. In the case of iron oxide and zinc oxide, this is facilitated by the energy transfer process [55]. The fluorescence of the molecule is quenched due to an energy transfer from the lowest excited singlet state to another electronic state of the metal, resulting in a loss of fluorescence [56].

### 2.4. PLGA Particles Incorporating Zinc Oxide and Iron Oxide: Analysis of Magnetic Resonance Imaging Properties

**Figure 4:**
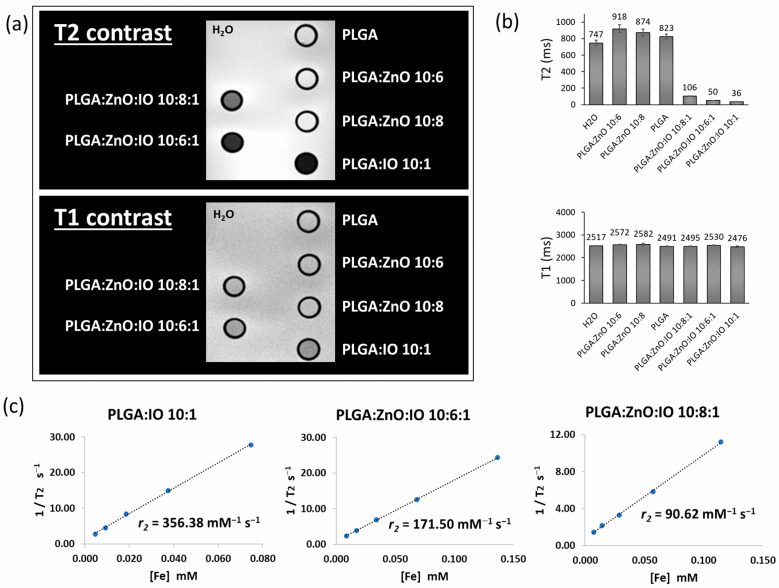
MRI characterization of PLGA samples: (**a**) *T*2-weighted (TE = 64 ms and TR = 2000 ms) and *T*1-weighting images (TE = 8 ms and TR = 400 ms) of PLGA samples acquired at 7T. The concentration of the samples is fixed at 2.5 mg mL^−1^; (**b**) *T*2 and *T*1 values of the PLGA samples (n = 3, mean ± SD); (**c**) relaxivity calculation for PLGA samples containing IO and ZnO:IO. The calculation was made based on the concentration of iron in each sample based on ICP results.

Magnetic resonance imaging (MRI) was used to evaluate the magnetic properties of PLGA particles containing ZnO and IO. Samples of PLGA:ZnO:IO 10:6:1 and 10:8:1 were analyzed as they exhibited the highest luminescence properties. The MRI images are shown in Figure 4.

To calculate the relaxivity for each sample, the concentration of Fe was measured by ICP (Table 3). The relaxivity value (*r*) was determined using the relaxivity linearity plot (Figure 4c), according to Equation (1):

(1) $$\frac{1}{T}=r\left[Fe\right]+\frac{1}{T_{0}}$$

where 1/*T* is the relaxation rate (inverse of the relaxation time *T*) in s^−1^, *r* is the relaxivity, $\left[Fe\right]$ is the iron concentration in mM, and 1/*T*_0_ is the intercept, representing the relaxation in the absence of iron. The relaxivity values are summarized in Table 3.

As shown in Figure 4a,b, an effect on the *T*2-weighted image is observed when IO is present (samples of PLGA:IO 10:1, PLGA:ZnO:IO 10:6:1, and PLGA:ZnO:IO 10:8:1), with the most significant effect observed for the PLGA containing only IO. Quantitative analysis of these images confirms a reduction in *T*2 values for PLGA:IO 10:1 (36 ± 1 ms) and PLGA:ZnO:IO 10:6:1 and 10:8:1 (50 ± 1 ms and 106 ± 1 ms, respectively) compared to unloaded PLGA (823 ± 25 ms). On the *T*1-weighted images, a very limited effect was observed, as is commonly seen with NPs [57,58]. The *T*2 and *T*1 results related to different concentrations of iron are shown in Table S1 and Figure S2.

The *r2* results (Figure 4c) demonstrate high relaxivity, which is probably associated with aggregation of IO NPs [57]. Interestingly, the presence of ZnO NPs negatively impacts the PLGA system’s relaxivity. This may be related to the organization of IO NPs within the PLGA particle, where the ZnO NP seems to limit the aggregation of IO, as observed in the TEM and SEM images presented in Figure 2 (Section 2.2). The addition of ZnO NPs can act as a spacer, reducing the dipolar interactions between the magnetic moments of IO NPs [59].

According to the ICP results shown in Table 3, it can be observed that the amount of zinc is approximately ten times higher than that of iron in moles. Additionally, this ratio increases with the nominal concentration of ZnO in the formulation (Fe:Zn, 1:10 to 1:13). Since the ratios can affect the aggregation state of the iron oxide NPs, this may explain why the *r2* value of the PLGA:ZnO:IO 10:8:1 formulation is lower than that of the PLGA:ZnO:IO 10:6:1 formulation.

### 2.5. PLGA Particles Incorporating Zinc Oxide and Iron Oxide: Analysis of Cytotoxicity and Cellular Labelling

**Figure 5:**
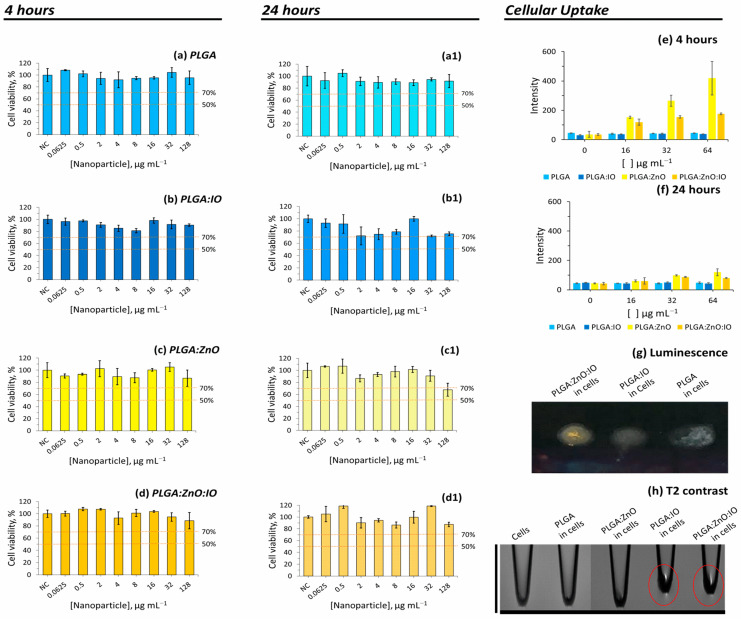
Resazurin cytotoxicity assay of PLGA particles: cell viability versus concentration of PLGA samples after incubating for 4 h (**a**–**d**) and 24 h (**a1**–**d1**) (*n* = 3, mean ± SD) (NC = negative control). Kruskal–Wallis followed by Dunn’s test compared with NC (*p* > 0.05); uptake of PLGA particles containing ZnO and IO NPs by cells assessed through luminescence assay at incubation times of 4 h (**e**) and 24 h (**f**), with an excitation wavelength of 343 nm and an emission wavelength of 540 nm (*n* = 3, mean ± SD). UV chamber luminescence (λ_ex_ = 245 nm) of uptake cells is shown in (**g**), and uptake of cells assessed by MRI test using *T*2 contrast (TR = 3000 ms and TE = 7.5 ms) is shown in (**h**).

THP-1 cells, a human monocytic cell line capable of differentiating into highly phagocytic macrophages, were used in this study. The cytotoxicity assay was performed with unloaded PLGA particles, as well as those loaded with IO, ZnO, or both, evaluating their effects as a function of particle concentration and incubation time (Figure 5). None of the tested systems induced a statistically significant reduction in cell viability, regardless of the dose or incubation period.

A cellular labelling assay was conducted to evaluate the ability of PLGA samples to mark THP-1 cells (Figure 5e–h). The labelling efficiency was evaluated after 4 h (Figure 5e) and 24 h (Figure 5f) of incubation, using luminescence testing based on the properties of ZnO.

After the 4 h incubation period, only the cells incubated with ZnO NP-containing PLGA were detectable using luminescence (Figure 5g), in a concentration-dependent manner (Figure 5e). The PLGA and PLGA-IO samples were used as controls to demonstrate the absence of fluorescence in cells without ZnO.

On the other hand, increasing the incubation time up to 24 h led to a reduction in luminescence (Figure 5e–g). One hypothesis to explain this observation is that, over time, the particles undergo cellular metabolism, where the acidic pH dissolves the luminescent ZnO, thus accounting for the loss of luminescence [60]. A study conducted by Lallo et al. (2022) revealed that ZnO behaves differently in more acidic pH environments, leading to its dissolution [53]. Another study by Cai et al. (2017) revealed that the endocytosis of tumor cells can dissolve ZnO when in contact with the lower pH of the cellular metabolism [61].

The cellular uptake assessed using MRI after 4h incubation (Figure 5h) showed that PLGA particles containing IO, such as PLGA:IO and PLGA:ZnO:IO, provided a strong *T*2 contrast, showing that the double-labelled PLGA particles were also detectable using MRI. These results indicate that this approach could be promising for biomedical imaging applications.

## 3. Materials and Methods

### 3.1. Materials

Zinc acetate dihydrate C_4_H_6_O_4_Zn.2H_2_O, iron (II) chloride hexahydrate 98%, iron (III) chloride hexahydrate 97%, tetramethylammonium hydroxide pentahydrate 97% (TMAOH), oleic acid 90%, perchloric acid, poly(lactic-co-glycolic acid) polymer (Resomer RG 503H, PLGA 50:50), Tween 80 (Polysorbate 80) (T80), triethylamine, dichloromethane (DCM), and polyvinylalkohol 20–98 (PVA) were purchased from Sigma-Aldrick^®^ (Saint Louis, MO, USA); lithium hydroxide monohydrate LiOH.H_2_O Chemicals™ (Ribeirão Preto, Brazil); ethanol 99.5%(Qhemis, São Paulo, Brazil); ethyl acetate 99.8% Fisher Chemical™ (Loughborough, UK). The water Milli-Q was obtained by an ultrapurification process in Merck™ Millipore (Trosly-Breuil, France).

### 3.2. Synthesis

#### 3.2.1. Zinc Oxide NPs

ZnO NPs were obtained by a sol-gel method according to Spanhel and Anderson (1991) [47], with some modifications. It consists of 2 main stages: (1) Formation of acetyl zinc acetate precursor: Zinc acetate hexahydrate and 50 mL of ethyl alcohol were added in a refluxing system at 98 °C for 2 h, obtaining a solution of 0.1 M. After this step, the solution was cooled at RT and diluted to a final concentration of 0.05 M. (2) Formation of the ZnO colloidal suspension: the colloidal suspension of ZnO was carried out by a hydrolysis and condensation reaction, in high power ultrasound (frequency 37, power 70%), at 60 °C, for 60 min, using lithium hydroxide (LiOH) as a hydrolysis agent. The 1:1 molar ratio of Zn:OH was used.

The coating method with oleic acid was performed following the procedure described by Manaia et al. (2015) [62], with some modifications. For that, 75 μL of oleic acid was added to 10 mL of the pre-prepared QD ethanolic suspension. The final concentration of oleic acid was 18.57 mM. The reaction was made at 30 min, up to 60 °C with stirring at 400 rpm. The suspension was stored overnight at RT (25 °C). The final suspension was washed 3 times with 10 mL of ethanol and centrifuged at 2000 rpm at RT. The centrifuged sample was diluted in DCM to a final concentration of 40 mg mL^−1^. The ZnO NPs were characterized by X-ray diffraction (XRD) and absorbance measurements, with detailed results presented in Figure S3.

#### 3.2.2. Superparamagnetic Iron Oxide NPs (SPIONs)

The synthesis of IO NPs was made according to Babes et al. (1999) [48], with some modifications. Solutions of 0.2 mL of FeCl_2_.4H_2_O 0.314 M and FeCl_3_.6H_2_O 0.666 M were added dropwise in 2 mL of 1 M TMAOH solution, magnetically stirred at RT for 5 min. Then, the solution was transferred to a 75 °C water bath, and 75 μL of oleic acid was added, stirred for 15 min, and stirred for 15 min at RT. The final solution was neutralized using 2 mL of HCLO_4_ 1 M, then washed 10 times with pure water and 3 times with ethanol PA. The final product was dried and dispersed in DCM in a concentration of 40 mg mL^−1^.

#### 3.2.3. PLGA Particles

The synthesis of the PLGA system dispersed in water was conducted following the procedure of a simple solvent evaporation emulsion reaction made by Luque-Michel et al. (2021) [45], with some modifications. Initially, 10 mg of PLGA (Resomer RG 503H) was dissolved in 0.8 mL of a mixture of triethylamine and acetyl acetate solution (1:1000). Subsequently, 0.2 mL of the contrast agent in DCM was added at the appropriate concentration. This solution was added dropwise into 2 mL of T80 1% under continuous agitation at RT. The mixture was sonicated using an ultrasonic probe system at 20 W for 20 s. The resulting emulsion was added dropwise to a solution of T80 0.3% and PVA 0.4%, and the mixture was kept under constant agitation for 1.5 h with the flask uncapped. The dispersion was then centrifuged and washed three times with pure water. The final product was dispersed in water containing trehalose 37% (a nominal amount based on trehalose:PLGA, *w*:*w*).

### 3.3. Characterization

#### 3.3.1. Evaluation of Luminescent Properties

The photoluminescence properties were evaluated by measuring the spectra of emission by photoluminescence (PL) and the excitation spectra by photoluminescence excitation (PLE). The spectra were recorded in the UV–visible range on a spectrophotometer Fluorolog™ 3 at RT, equipped with a double excitation monochromator, and a Hamamatsu™ R928 photomultiplier with a Xe lamp (450 W). The wavelength of emission and excitation was based on the previous wavelength test.

#### 3.3.2. Transmission Electron Microscopy

TEM images were obtained with a JEOL Electron Microscope (model JEM-1400; SUPER TWIN) (JOEL Ltd., Tokyo, Japan) operated at 600 kV of 60 magnitude. The samples dispersed in water were deposited on carbon grids. Image analysis was performed by ImageJ software (version 1.53t, National Institute of Health, Bethesda, MD, USA) with 20 and 50 nm scale bars.

#### 3.3.3. Scanning Electron Microscopy and Energy-Dispersive X-Ray Spectroscopy (EDS)

SEM was carried out to evaluate the visual aspects of the nanocrystals. The samples were carbon-coated to about 15 nm thickness using a Sample Sputter Coater SCD 050™ (Bal-Tec, Wallruf, Germany) for 70 s. After that, SEM pictures were taken using a scanning electron microscope SM300™ (TOPCON, Singapore) instrument.

Additionally, the elemental composition was determined using energy-dispersive X-ray spectroscopy (EDS) attached to the SEM. EDS spectra were obtained from selected areas of the samples, enabling qualitative and semi-quantitative identification of the elements present in the NP. The data were analyzed using JSM-IT500HR (JEOL ltd., Tokyo, Japan), providing information on the elemental distribution across the particle.

#### 3.3.4. Inductively Coupled Plasma Mass Spectrometry (ICP-MS)

Iron and zinc concentrations were measured using inductively coupled plasma mass spectrometry (ICP-MS), following the protocol established in collaboration with the Institute of Chemical Sciences of the University of Rennes (ISCR).

#### 3.3.5. Evaluation of Particle Diameter Distribution and Zeta Potential

Particle diameter distribution and zeta potential measurements were performed using a Malvern Dynamic Light Scattering (DLS) system (Malvern™, Malvern, UK). The samples were diluted in distilled water to achieve an optimal count rate (>300 kcps) and attenuation level (attn) between 6 and 9, following the equipment’s operational parameters. The dilution factor was adjusted empirically to ensure reliable measurements. All measurements were conducted at 25 °C, and the results were collected in triplicate.

#### 3.3.6. Magnetic Resonance Imaging

The magnetic resonance imaging (MRI) was carried out using a 7T scanner (Biospec 70/20 Avance III, Bruker™, Wissembourg, France) equipped with a BGA12S gradient system (675 mT/m) applying a magnetic field of 7 Tesla at room temperature. Emission and reception were ensured by a 72 mm diameter resonator. Sets of spin echo sequences weighted in *T*1 or *T*2 were acquired using variable repetition times (from 200 to 10,000 ms), with a fixed echo time of 8 ms and a fixed repetition time of 2000 ms for variable echo times (ranging from 8 to 200 ms). In both cases, images were acquired with a field of view = 6 cm × 4 cm, a matrix = 256 × 192, and a slice thickness of 2 mm.

### 3.4. Biological Analysis

#### 3.4.1. Cytotoxicity Assay Using Resazurin Test

The cell uptake assay was made using the THP-1 human monocytic cell line, which was obtained from ATCC, catalog number TIB-202. The differentiation of THP-1 cells into macrophage-like cells was induced by adding phorbol 12-myristate-13-acetate (PMA, Sigma-Aldrich™, Burlington, MA, USA) at a concentration of 100 nM at the time of seeding (time 0). The cells were incubated with PMA for 48 h before further experiments.

The cells were plated in a 96-well plate with a seeding density of 8.5 × 10^3^ cells per well. After 24 h, PLGA NPs were introduced at concentrations ranging from 0.078 to 128 μg mL^−1^, followed by a 4 h and 24 h incubation period. Subsequently, the cells were washed and allowed to grow for an additional 24 h before the addition of 20 μL of a 25 μg mL^−1^ resazurin solution in phosphate-buffered saline (PBS). After 4 h, the wells were analyzed using the EnSpire™ microplate reader (PerkinElmer, Waltham, MA, USA) at 570 nm wavelength excitation, and the baseline emission was corrected at 590 nm. The percentage of viable cells was calculated using Equation (2):

(2) $$Viable\;cells\;\left(\%\right)=\frac{\left(O.D.\;sample-O.D.\;media\right)}{\left(O.D.\;cells-O.D.\;media\right)}\times 100$$

where viable cells (%) represent the percentage of living cells in the sample, *O.D. sample* is the optical density of the sample with cells, O.D. media is the optical density of the cell-free medium, and O.D. cells is the optical density of a sample with a known number of viable cells. The analyses were conducted in triplicate. A statistical analysis was carried out using the Kruskal–Wallis test followed by Dunn’s test, to compare the viability between the concentrations of PLGA samples and the control, as well as between different incubation times. A significance level of 0.05 was considered for all analyses. Data processing and statistical tests were conducted using GraphPad Prism 9.

#### 3.4.2. Cellular Uptake

The cellular uptake assay was performed using the TPH-1 cell line. The cells were seeded onto a 24-well plate with a seeding density of 8.5 × 10^3^ cells per well. The concentration between 16 and 64 µg mL^−1^ was tested according to the cytotoxicity assay for the different samples. Incubation times were fixed at 4 and 24 h. After the incubation, the cells were washed to remove the NPs not captured by the cells. Fluorescence analyses were carried out using EnSpire™ microplate reader (PerkinElmer, Waltham, MA, USA), with excitation at 343 nm and emission at 540 nm. MRI analyses of the cells were performed according to the procedures outlined in Section 3.3.6, following a 4 h incubation period.

## 4. Conclusions

In this study, we present a nanoscale bimodal contrast agent for fluorescence and magnetic resonance imaging, formulated using poly(lactic-co-glycolic acid) (PLGA) encapsulating zinc oxide (ZnO) and iron oxide (IO) nanoparticles. Before the biological assessment on THP-1 cells, an extensive investigation into the interaction of these oxides and their impact on imaging properties was conducted. Our findings demonstrate the potential of PLGA:ZnO:IO particles for biomedical applications; this is attributed to their combined luminescent and magnetic properties. This bifunctional nanosystem holds promise for advanced imaging modalities in biomedical research and clinical diagnostics.

## Figures and Tables

**Table 1:** Stability study of PLGA suspensions after particle synthesis and following a 15-day storage period in suspension. DLS analysis.

**PLGA:IO** **(** ***w*** **:** ***w*** **, mg)**	**Synthesis**		**15 Days**	
	**Z-Average** **(d.nm)**	**PDI**	**Z-Average** **(d.nm)**	**PDI**
10:1	152.1 ± 0.907	0.099 ± 0.026	149.5 ± 0.411	0.120 ± 0.015
10:2	146.8 ± 0.800	0.128 ± 0.013	144.9 ± 3.107	0.174 ± 0.024
10:3	148.7 ± 1.457	0.106 ± 0.007	148.1 ± 0.818	0.136 ± 0.014
10:4	147.8 ± 0.568	0.184 ± 0.017	145.4 ± 2.931	0.164 ± 0.010
**PLGA:ZnO (*w*:*w*, mg)**	**Synthesis**		**15 days**	
	**Z-average** **(d.nm)**	**PDI**	**Z-average** **(d.nm)**	**PDI**
10:2	251.5 ± 1.473	0.118 ± 0.016	243.0 ± 1.082	0.096 ± 0.022
10:4	211.4 ± 10.95	0.205 ± 0.014	254.2 ± 1.950	0.189 ± 0.023
10:6	224.6 ± 2.948	0.078 ± 0.040	226.3 ± 1.952	0.069 ± 0.025
10:8	219.4 ± 1.266	0.093 ± 0.016	214.8 ± 1.450	0.095 ± 0.002
**PLGA:ZnO:IO (*w*:*w*:*w*, mg)**	**Synthesis**		**15 days**	
	**Z-average** **(d.nm)**	**PDI**	**Z-average** **(d.nm)**	**PDI**
10:8:1	247.0 ± 2.254	0.157 ± 0.020	236.0 ± 2.227	0.144 ± 0.019
10:8:2	280.3 ± 2.887	0.204 ± 0.042	270.1 ± 5.508	0.191 ± 0.022
10:8:3	236.1 ± 1.589	0.233 ± 0.006	277.1 ± 9.296	0.289 ± 0.109
10:8:4	264.6 ± 7.534	0.288 ± 0.034	318.6 ± 69.12	0.409 ± 0.113
**PLGA:ZnO:IO (*w*:*w*:*w*, mg)**	**Synthesis**		**15 days**	
	**Z-average** **(d.nm)**	**PDI**	**Z-average** **(d.nm)**	**PDI**
10:4:1	193.8 ± 0.265	0.061 ± 0.012	200.1 ± 0.448	0.091 ± 0.017
10:6:1	235.0 ± 0.758	0.066 ± 0.021	222.0 ± 1.778	0.096 ± 0.030
10:8:1	247.0 ± 2.254	0.157 ± 0.020	236.0 ± 2.227	0.144 ± 0.019

**Table 2:** The average diameter of ZnO and IO was analyzed by TEM and calculated using a Gaussian curve, while the zeta potential of the PLGA systems in water was measured before the lyophilization process.

PLGA:ZnO:IO	Average Diameter NPs (nm)	Zeta Potential
10:0:0	-	−21.3 ± 1.0
10:6:0 (ZnO)	4.73 ± 0.16	−12.2 ± 1.6
10:8:0 (ZnO)	4.56 ± 0.16	−17.5 ± 0.8
10:0:1 (IO)	7.33 ± 0.28	−25.4 ± 0.2
10:6:1 (ZnO + IO)	4.03 ± 0.10	−33.2 ± 1.4
10:8:1 (ZnO + IO)	4.64 ± 0.14	−31.9 ± 1.0

**Table 3:** Relaxivity (*r2*) of PLGA:ZnO:IO samples, followed by ICP results of Zn and Fe elements and their ratio.

Samples	Fe (µM)	Zn (µM)	Fe:Zn (mol:mol)	*r2* (mM^−1^ s^−1^)
PLGA:IO 10:1	4.15 ± 0.33	-	-	356.38
PLGA:ZnO:IO 10:6:1	4.98 ± 0.26	50.01 ± 0.34	1:10	171.50
PLGA:ZnO:IO 10:8:1	3.44 ± 0.20	45.57 ± 0.42	1:13	90.62

## Data Availability

Data will be made available on request.

## Supplementary Materials

The following supporting information can be downloaded at: https://www.mdpi.com/article/10.3390/molecules30081818/s1, Figure S1. TEM analyses of IO NPs; Table S1. Results of *T*2 and *T*1 in different concentrations of PLGA systems; Figure S2. Relaxivity values (r1 and r2); Figure S3. Characterization of ZnO NP [63,64,65].

## Abbreviations

The following abbreviations are used in this manuscript:

DCM	Dichloromethane
EDS	Energy-Dispersive Spectroscopy
IO	Iron Oxide
MRI	Magnetic Resonance Imaging
NC	Negative Control
NP	Nanoparticle
PDI	Polydispersity Index
PLGA	Poly(lactic-co-glycolic acid)
PVA	Polyvinyl Alcohol
RT	Room Temperature
SEM	Scanning Electronic Microscopy
SPION	Superparamagnetic Iron Oxide Nanoparticles
T80	Tween 80
TEM	Transmission Electronic Microscopy
TE	Echo Time
THP-1	(Human monocytic cell line)
TR	Repetition Time
ZnO	Zinc Oxide
