# Supplementary material for: Bimodal Poly(lactic-co-glycolic acid) Nanocarrier with Zinc Oxide and Iron Oxide for Fluorescence and Magnetic Resonance Imaging
Source: Molecules. 2025 Apr 18;30(8):1818. doi: 10.3390/molecules30081818 (PMC12029633; doi:10.3390/molecules30081818)
Supplement: Supplementary file 1 [file molecules-30-01818-s001.zip › molecules-3527239-supplementary.pdf]

Bimodal Poly(lactic-co-glycolic acid) Nanocarrier with Zinc Oxide and Iron Oxide for Fluorescence and Magnetic Resonance Imaging.

Thúlio Wliandon Lemos Barbosa[1,2,*], Laurent Lemaire[2,3,*], Isabelle Verdu[2], Larissa Santos[1], Mariana Picchi Salto[1], Natália Galvão de Feritas[1], Leila Aparecida Chiavacci[1*].

1. School of Pharmaceutical Sciences, São Paulo State University (UNESP), 14800-903 Araraquara, SP, Brazil; l.santos@unesp.br (L.S.); n.freitas@unesp.br (N.G.d.F.); mariana.psalto@gmail.com (M.P.S.).

2. National Institute of Health and Medical Research (INSERM), National Centre for Scientific Research (CNRS), Materials, Engineering, Nanosciences, and Technologies (MINT), Research and Training System - Interac-tions, Catalysis, Applications, and Technologies (SFR ICAT), University of Angers, F49000 Angers, France; isabelle.verdu@univ-angers.fr (I.V.)

3. Platform for Research in Imaging and Multimodal Spectroscopy (PRISM), SFR ICAT, University of Angers, F49000 Angers, France.

*Correspondence: thulio.lemos@unesp.br (T.W.L.B.); laurent.lemaire@univ-angers.fr (L.L.); leilachiavac-ci@unesp.br (L.A.C.)

Supplementary Material

Figure S1. TEM analyses of IO NPs

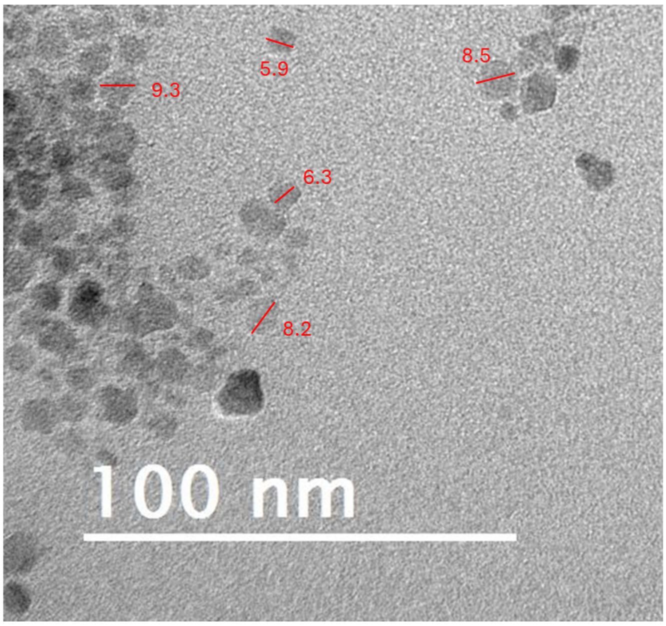

Average diameter of 8.07 nm.

Table S1 Results of *T2* and *T1* in different concentration of PLGA systems.

PLGA:IO 10:1			PLGA:ZnO:IO 10:6:1			PLGA:ZnO:IO 10:8:1		
[Fe] mM	1/T2	1/T1	[Fe] mM	1/T2	1/T1	[Fe] mM	1/T2	1/T1
0,07	27,78	0,39	0,14	24,39	0,39	0,11	11,24	0,38
0,04	14,93	0,39	0,07	12,50	0,39	0,06	5,81	0,37
0,02	8,33	0,40	0,03	6,90	0,40	0,03	3,28	0,37
0,01	4,50	0,39	0,02	3,80	0,39	0,01	2,17	0,38
0,00	2,72	0,39	0,01	2,35	0,39	0,01	1,47	0,37

Figure S2. Relaxivity values (r1 and r2).

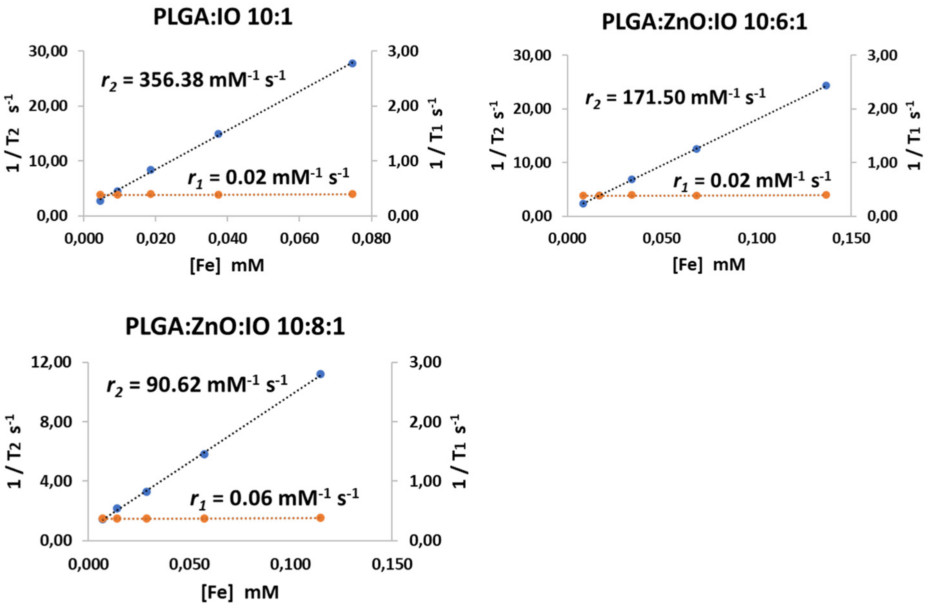

No effect on *r1* was observed in the analyses. The results of *r2* were discussed in the text.

Figure S3. Characterization of ZnO NP.

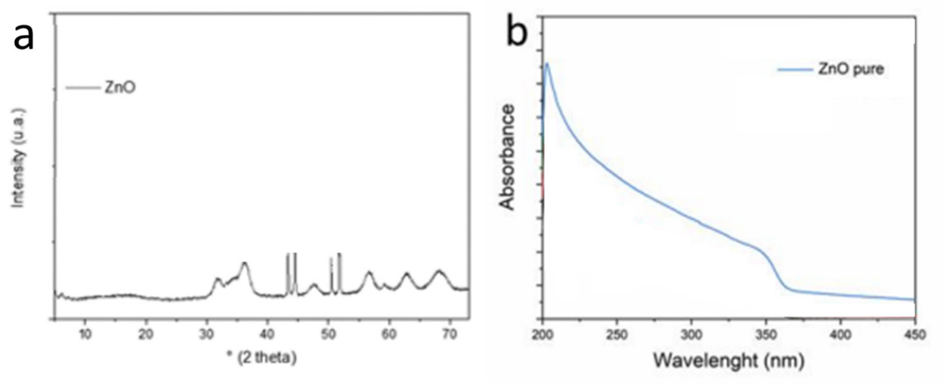

a. Some additional peaks appeared in the X-ray diffractogram of the samples; however, these peaks located at 43.32°, 44.46°, 50.43°, and 51.77° 2θ angles result from the metallic apparatus used for conducting the experiments. X-ray diffraction pattern shows the presence of the ZnO phase. The Bragg peaks found were 2θ = 31.81°, 34.09°, 36.30°, 47.80°, 56.83°, 62.83°, and 68.29°, which correspond to the (100), (002), (101), (102), (110), (103), and (112) planes of ZnO crystal identified in ZnO sample (BA-ABBAD et al., 2013; SEKAR et al., 2018)[63,64].

b. The absorbance plots of the sample display the ZnO band at ~350 nm [65].

63. Ba-Abbad, M.M.; Kadhum, A.A.H.; Mohamad, A.B.; Takriff, M.S.; Sopian, K. Visible Light Photocatalytic Activity of Fe3+-Doped ZnO Nanoparticle Prepared via Sol-Gel Technique. *Chemosphere* 2013, *91*, 1604–1611, doi:10.1016/j.chemosphere.2012.12.055.

64. Sekar, A.D.; Muthukumar, H.; Chandrasekaran, N.I.; Matheswaran, M. Photocatalytic Degradation of Naphthalene Using Calcined Fe–ZnO/ PVA Nanofibers. *Chemosphere* 2018, *205*, 610–617, doi:10.1016/j.chemosphere.2018.04.131.

65. Krobthong, S.; Wongrerkdee, S.; Pimpang, P.; Moungsrijun, S.; Sujinnapram, S.; Nilphai, S.; Rungsawang, T.; Wongrerkdee, S. ZnO Nanoparticles Coprecipitation with Aluminum and Copper Ions for Efficient Photocatalytic Degradation of Commercial Glyphosate. *Integrated Ferroelectrics* 2022, *222*, 69–83, doi:10.1080/10584587.2021.1961517.
